# Chicago Medical Society

**Published:** 1869-01

**Authors:** 


### CHICAGO MEDICAL SOCIETY.

NOVEMBER 20, 1868.

The Society was called to order by the President, Dr. Marguerat, after which the Secretary read the proceedings of the last meeting.

Under call for Pathological Specimens, Dr. Fisher read a report, and presented for the examination of the Society the kidneys of a patient who died November 18. The case presented the symptoms of general dropsy, urine albuminous; and tapping was resorted to several times. After tapping, the amount of urine passed was very much increased. In July last, the patient had an attack of pleurisy, the bloating continuing. Liver was enlarged. Mitral regurtitation. A *post mortem* was held by Drs. Fisher, Bogue, and others. Found about a gallon of fluid in the abdominal cavity, two quarts in thorax, and about four ounces in the pericardium. The heart was hypertrophied. Liver enlarged, and uneven. The kidneys presented were fibrinous, atrophied, and contained an earthy concretion in the centre.

Dr. Fisher asked if any member had noticed, after tapping, this extensive passage of urine (two gallons in 24 hours)?

Dr. Reid said he had never seen such a case, but had noticed a great increase in the amount of urine voided after labor, and probably due to same cause; but, instead of the fluid, the fœtus compressing and congesting the kidneys.

Dr. Davis asked if this condition of the kidneys and liver were not due to rheumatism or cardiac disease? The Doctor was inclined to think it due to the latter. Remarked that he could recall several cases of chronic rheumatism which have existed ten or twelve years without showing albumen in the urine. Thinks, had the urine been examined before the cardiac difficulty, no albumen would have been found. Thinks the excessive urination after tapping was not of frequent occurrence. Cited case corner Burnside and 18th Streets.

Dr. Paoli presented a plate showing a case of Elephantiasis Græcorum, from a man whom he had seen in New York City. The patient came from Europe, after suffering three years, hearing the climate would probably prove beneficial. Also presented a patient to the Society whose arm and face showed signs of the same disease, and whose sister had elephantiasis.

Dr. Reid said he never should have known that the patient presented was suffering from elephantiasis, had he not been so informed by Dr. Paoli. That it was a new form to him, and that he supposed there was great enlargement, and that it was incurable.

Dr. Fredigke presented drawings representing a case of "Ectropium of the Intestines." The child was born November 15th, in the 8th month of gestation. The umbilicus was perfectly formed, and the child lived 21½ hours.

Dr. Paoli remarked, that he presented a similar case some years ago, the child being about 7 months old, and being born with but one arm.

The Society proceeded to discuss the subject as to the "Prevalence of Typhus and other Fevers," which was chosen at a previous meeting.

Dr. Davis opened the discussion, by relating somewhat in detail the characteristic symptoms of several cases of continued fever, as samples of a considerable number of cases that had come under his observation during the last two months. They differed from ordinary cases of typhoid fever, in attacking many children; in attacking several successively in the same family, and presenting but little tendency to diarrhœa. They also occurred mostly among the poor, in bad sanitary localities.

Dr. Clarke remarked, that he did not think we were having typhus fever; at least, nearly all that came under his observation were intermittent and remittent, and an occasional case of typho-malarial fever. Cited case of a fellow-practitioner, who saw some cases which had a queer look. Was called in case of a child six years old. Found it to be a case of purely intermittent. Gave quinine, and child recovered in about eight days.

Dr. Paoli remarked, that he had not seen a case of typhus fever in Chicago for eight years. Thinks cases reported by Dr. Davis are similar to typhus, but wishes to know whether they are typhus or typhoid. Said we had cases of typhoid very similar to typhus, attended with great emaciation, with the eruption more on the extremities than abdomen.

Dr. Foster asked on what day the eruption occurred in these fevers, and their difference?

Dr. Marguerat reported a case where there was no diarrhœa, the fever continuing three weeks; the patient dying the latter part of the fourth week, from brain symptoms. Remarked, that Dr. Dyas reported two cases of typhus fever to the Board of Health some three months ago.

Dr. Davis said, that the time of the appearance of the eruption was variable, and in cases of some children there was none. Cited case in hospital, who was admitted fourth or fifth day after onset. Noticed the eruption the next day after admission, in form of a fine red papule. Not ecchymosis, but a simple exanthematous rash, appearing in all cases, from the sixth to the tenth day.

Does not find the eruption in typhus in all cases. Considers the difference between the typhus and typhoid to consist in the absence of tympanitis and loose bowels which exist in the latter. Cited case in Mercy Hospital, upon which he held a clinic October 7, which he thought to be typhus instead of typhoid. Also reported a case of typhus continuing nine or ten days, resulting in death. Remarked, that it was not his object to maintain that we had typhus in the city, but to elicit the experience of the Society. Asked the Society if any one had seen cases of cerebro-spinal meningitis or spotted fever? Reported several deaths where patients were attacked suddenly. No convulsions, no opisthotonos, but constant delirium.

Dr. Marguerat said he was called where three children died suddenly, but he believed the disease to be scarlet fever.

Dr. Wickersham remarked, that he had seen a great deal of scarlet fever of late, and he had no doubt but what the cases dying off suddenly were those of scarlet fever.

Dr. Davis said, that in those cases occurring on Clinton Street, a practitioner on the West Side said it was scarlet fever.

The subject chosen for next discussion of Society was, "Would "it be conducive to Morality to limit the Diseases arising from "Prostitution?"

Drs. Bridge and Gray were appointed to open discussion.

On motion, Society adjourned.

---

NOVEMBER 27, 1868.

The Regular Meeting of the Society, in the absence of the President, was called to order by the Vice-President, Dr. Bogue.

The Secretary then read the proceedings of the last meeting, which were approved, and ordered to be placed on file.

Under the call for Pathological Specimens, Dr. Bogue presented for the inspection of the Society a part of the lower jaw of a man some 20 or 25 years of age, which was the seat of a medullary or internal carcinomatous tumor, removed that day by Dr. Powell, at the County Hospital.

The tumor first appeared some 20 months ago, and, as a rule, the patient has suffered but little pain. There were no glandular enlargements about the mouth; and, taking these matters into consideration, Dr. Bogue said that he did not think the growth malignant.

The operation consisted in removing a tooth at the point where the division was to be made; then making an incision along the inferior angle of the jaw; after which, the chain-saw was applied, and division made. An instrument was then used to divest the portion of the jaw of the periosteum, when the jaw was pulled out. The facial artery was the only one ligated in the operation. Dr. Powell thinks that where the portion of the jaw is pulled out instead of being dissected out, there is decidedly less hemorrhage.

Dr. Ross presented the heart of a woman 44 years of age, who died in County Hospital, showing a large aneurism of the aorta. The patient has been under treatment altogether about

18 months, and for the past two months, and up to time of death, in the County Hospital. The Doctor states, that when he was called to see the patient, she was in a fainting-fit, which was followed by difficulty in breathing. The pulse was small, frequent, and patient complained of weight in chest. If in the semi-recumbent position on left side, she felt quite comfortable; but upon turning on the right side, she was troubled with great dyspnœa.

On next morning, patient was better. Pulse regular, although at times there was difficulty in swallowing. Respiration accompanied by a wheezing, laryngeal sound. Valvular sounds of heart normal. Tongue moist and clean.

About October 1, commenced giving iodide and bromide of potassa, chloric ether, tinct. verat. viride, and tinct. iron; which treatment was continued about four weeks. Voice broken, as though the vocal cords were relaxed.

October 28, Dr. Davis was called in counsel. From this time patient kept her bed. There was detected a wavy impulse one inch to right of sternum, between the first and second ribs. Heart-sounds continued natural.

November 21, patient died, with symptoms of great dyspnœa.

*Post Mortem.*—Found left lung somewhat collapsed, and both emphysematous.

Dr. Ross remarked, that he had never seen a case where so large an aneurism existed, where the subclavian and carotid arteries were not blocked up by coagula.

The leading symptoms throughout the entire course of the disease were dyspnœa and a wheezing cough.

Dr. Ross said he could not account for patient's not being able to lie on right side. Respiratory murmur good all over chest. No dulness on percussion. Remarked, that deceased has a sister living on the North Side, who probably has fatty degeneration of the heart.

Dr. Gray reported a case of Angina Pectoris, which proved fatal in twenty minutes.

Dr. Clarke reported a case of Ovariotomy—Mrs. H., of Fort Atkinson, Ohio. The tumor was removed August 6, 1867.

The patient made a rapid recovery, and is now in the enjoyment of good health. Drs. Davis and Bickney, of Fort Atkinson, under whose care the patient had been, assisted in the operation.

Drs. Paoli and Powell made some remarks on the case, the latter recommending silver sutures in ligating the pedicle, as he had seen one case where the discharge was kept up for a long time where silk was used, and finally resulting in a fistula.

Dr. Fitch said he had under treatment a case of Fibrous Tumor, which has been growing seven years. Patient suffers no inconvenience, except from weight of tumor. She has occasional attacks of cystitis, urethritis, or vaginitis. Had two attacks during past 18 months. Does not interfere with general health. Three years ago, patient was pale and anæmic. Gave syr. ferri iodidi, which has been continued most of the time since. At one time she omitted it for about three months, when she began to grow anæmic again.

Society adjourned.

